# Novel TRIM Proteins Modulating the Innate Immune Response of Rainbow Trout (*Oncorhynchus mykiss*)

**DOI:** 10.3390/ani16010076

**Published:** 2025-12-26

**Authors:** Francisco Donoso, Felipe Ramírez-Cepeda, Nicolás Salinas-Parra, Claudio A. Álvarez, Paula Santana, Rubén Avendaño-Herrera, Rafael Diego Rosa, Cristian A. Valenzuela, Byron Morales-Lange, Luis Mercado

**Affiliations:** 1 Department of Anatomy and Neuroscience, APC Microbiome Ireland, University College Cork, T12 YT20 Cork, Ireland; francisco.donoso@ucc.ie; 2 Grupo de Marcadores Inmunológicos, Laboratorio de Genética e Inmunología Molecular, Instituto de Biología, Pontificia Universidad Católica de Valparaíso, Valparaíso 2340000, Chile; felipe.ramirez@pucv.cl (F.R.-C.); nicolas.salinas@pucv.cl (N.S.-P.); cristian.valenzuela@pucv.cl (C.A.V.); 3 Laboratorio de Tecnología de Cultivo de Peces Marinos (TECPEMAR), Departamento de Acuicultura, Facultad de Ciencias del Mar, Universidad Católica del Norte, Coquimbo 1780000, Chile; claudio.alvarez@ucn.cl; 4 Instituto de Ciencias Aplicadas, Facultad de Ingeniería, Universidad Autónoma de Chile, Santiago 8580640, Chile; paula.santana@uautonoma.cl; 5 Laboratorio de Patología de Organismos Acuáticos y Biotecnología Acuícola, Facultad de Ciencias de la Vida, Universidad Andrés Bello, Viña del Mar 2340000, Chile; ravendano@unab.cl; 6 Interdisciplinary Center for Aquaculture Research (INCAR), Viña del Mar 2531015, Chile; 7 Centro de Investigación Marina Quintay (CIMARQ), Universidad Andrés Bello, Quintay 2340000, Chile; 8 LaBIOMARIS—Laboratory of Biotechnology and Marine Health, Department of Cell Biology, Embryology and Genetics, Federal University of Santa Catarina, Florianópolis 88035-972, Brazil; rafael.d.rosa@ufsc.br; 9 Department of Animal and Aquaculture Sciences, Faculty of Biosciences, Norwegian University of Life Sciences, 1432 Ås, Norway

**Keywords:** *Oncorhynchus mykiss*, Tripartite motif-containing proteins, fish innate immunology, PAMP’s signalling, LPS, poly I:C, *Flavobacterium psychrophilum*

## Abstract

In higher vertebrates, Tripartite Motif (TRIM) proteins modulate the immune response by coordinating processes related to inflammation such as antiviral restriction, autophagy and inflammasome activation. In fish, TRIM proteins have been reported mainly in cyprinids (e.g., carp—*Cyprinus carpio* and zebrafish—*Danio rerio*) and salmonids (i.e., rainbow trout—*Oncorhynchus mykiss*). However, their molecular mechanisms and functions are still being described in aquatic animals. Thus, our study focused on characterizing novel TRIM proteins involved in the innate immunity of gill cells from rainbow trout (RTgill-W1 and primary cultures) stimulated with lipopolysaccharide (LPS) or polyinosinic–polycytidylic acid (poly I:C). Furthermore, an *in vivo* experiment with rainbow trout was performed to detect TRIM proteins after the challenge with *Flavobacterium psychrophilum* (a major bacterial pathogen affecting Chilean salmonid industry). *In vitro* results showed that OmTRIM25 triggered an LPS-induced expression of pro-inflammatory cytokines such as TNF-α2 and IL-1β. Moreover, in the fish experiment, OmTRIM25 and finTRIM2 were up-regulated in the gills two days post-infection (dpi), whereas IL-1β and TNF-α2 had a higher gene expression at four and six dpi, respectively. To investigate the immunological role of OmTRIM25, a gene silencing strategy using RNA interference (RNAi) was used, confirming the immunomodulatory function of OmTRIM25.

## Simple Summary

This study investigates the role of Tripartite Motif (TRIM) proteins at *in vitro* and *in vivo* levels in gills from rainbow trout (*Oncorhynchus mykiss*). In mammals, TRIM proteins are known for coordinating immune processes like antiviral defence, autophagy and inflammasome activation. However, TRIM function is still under investigation in fish. Contributing to the knowledge of these proteins, our data showed that OmTRIM25 (identified as a novel TRIM protein) had an immunomodulatory role by promoting the expression of key pro-inflammatory cytokines (i.e., TNF-α2 and IL-1β) linked to the innate immune response after challenges.

## 1. Introduction

The study of cellular subpopulations and molecular components involved in the physiological response has provided important insights into the evolution of the immune system across vertebrates [1,2,3,4,5]. For instance, the Tripartite Motif (TRIM) protein family (a group with over 70 proteins in mammals) has been associated with several biological processes, including apoptosis, cellular proliferation and differentiation, as well as ontogenetic development (among others) [6,7]. TRIM proteins consist of an N-terminal RING finger domain, followed by one or two B-box zinc finger domains and a coiled-coil region, which are collectively known as the RBCC motif [8]. The RBCC motif is fairly conserved across the TRIM protein family, with more evident differences and specific functions linked to the less conserved C-terminal domain [9]. Also, the accumulating evidence suggests that TRIM proteins have an important role in the innate immune response [10,11], with a crucial impact in the modulation of pro-inflammatory mechanisms [12], such as regulation of antiviral restriction [13], cellular autophagy [14] and inflammasome activation [15]. For this reason, TRIM proteins could also be considered as intracellular cytokines. However, further information about their function and evolution in the immune system is needed to describe molecular features involved in host defence.

Over the last decade, advances have been made in the discovery and characterization of TRIM proteins related to innate immunity in fish models [16,17,18,19]. Indeed, several TRIM members found in mammals have already been confirmed to be expressed as orthologues in different fish. For example, this includes TRIM32 and TRIM47 in carp (*Cyprinus carpio*) [20,21] and TRIM69 in zebrafish (*Danio rerio*) [22]. Furthermore, a large new subset of TRIM genes has been specifically identified in rainbow trout (*Oncorhynchus mykiss*) and zebrafish [23]. These TRIM proteins exclusively identified in fish were called “novel fish TRIMs” or “finTRIMs”. However, their immunomodulatory mechanisms are still being investigated in cellular and animal models [17,24,25]. Recent research has shown that TRIM16 and TRIM65 proteins from pufferfish (*Takifugu obscurus*) play a fundamental role in defence against *Vibrio harveyi* through the modulation of autophagy-mediated immunity [26], whereas TRIM38 from Large yellow croaker (*Larimichthys crocea*) is involved in the immune response to cope with *Pseudomonas plecoglossicida* [27]. Thus, the literature proposes an important role of TRIM proteins against Gram-negative bacterial pathogens in fish. On the other hand, TRIM proteins have also been described as key components during antiviral responses. For instance, this includes TRIM59 from *Epinephelus akaara*, TRIM44 from *Siniperca chuatsi*, TRIM103 from *Ctenopharyngodon idella* and TRIM32 from *O. mykiss*) [28,29,30,31]. Another example is TRIM47 of *C. idella*, which deploys a ubiquitin-independent antiviral pathway via autophagy-mediated clearance, dismantling viral replication factories and inhibiting infection [32].

Considering this background, the purpose of the present study was to explore and characterize the presence of novel TRIM proteins involved in innate immunity in rainbow trout through the combination of *in vitro* and *in vivo* strategies. To achieve this, we identified and sequenced novel TRIM transcripts in the RTgill-W1 cell line (from rainbow trout gills). Then, TRIM expression patterns were studied both in RTgill-W1 cells and rainbow trout primary cultures following a stimulation with lipopolysaccharide (LPS), a cell wall component of Gram-negative bacteria, or with polyinosinic–polycytidylic acid (poly I:C), a molecule structurally like viral double-stranded RNA (dsRNA). Furthermore, we examined the time-dependent expression of TRIM proteins at *in vivo* level (in rainbow trout gills) after a challenge with *Flavobacterium psychrophilum*, a Gram-negative bacterial pathogen for salmonids. These approaches were coupled with post-transcriptional knock-down assays to determine the immunomodulatory functions of novel TRIM proteins on pro-inflammatory cytokines. Overall, the results provide fundamental insights into the implications of TRIM proteins in the antimicrobial defence of fish and shed light on the potential conserved mechanisms that these molecules may use to modulate the innate immune response in vertebrates.

## 2. Materials and Methods

### 2.1. Chemical and Reagents

L-15 medium was obtained from Life Technologies (Thermo Fisher Scientific, Waltham, MA, USA). Foetal bovine serum (FBS) was obtained from Biological Industries (Cromwell, CT, USA). Penicillin/streptomycin was obtained from Gibco (Thermo Fisher Scientific). Poly I:C was purchased from Merck (former Sigma-Aldrich, Darmstadt, Germany). LPS from *Pseudomonas aeruginosa* was provided by Prof. Alejandro Dinamarca (Universidad de Valparaíso, Valparaíso, Chile). FuGENE^®^ was obtained from Promega (Madison, WI, USA), and TRIzol^®^ was obtained from Thermo Fisher Scientific.

### 2.2. Culturing of RTgill-W1 Cells and Treatment

The gill epithelial cell line RTgill-W1 (ATCC^®^ CRL-2523™) from rainbow trout was gently provided by Prof. Brian Dixon (University of Waterloo, Canada) and cultured as previously described by Álvarez et al. [33]. Briefly, 6 × 10^5^ RTgill-W1 cells per well (6-well plates) were maintained in L-15 medium with 4 mM glutamine and supplemented with 5% FBS, 200 U mL^−1^ penicillin and 200 µg mL^−1^ streptomycin at 20 °C. Then, the cells were treated with 10 µg mL^−1^ LPS in FuGENE^®^ for 4, 8 and 24 h or with 30 µg mL^−1^ poly I:C in FuGENE^®^ for 4, 12 and 24 h. Induction periods and LPS and poly I:C doses were selected according to gene expression patterns (related to inflammatory response) previously observed in RTgill-W1 cells [33].

### 2.3. Primary Culturing of Rainbow Trout Gill Cells

Pre-smolts rainbow trout (40–50 g) were obtained from Río Blanco fish farm (Pontificia Universidad Católica de Valparaíso), a certified pathogen-free hatchery located in Los Andes, Chile [34]. Fish were sacrificed using an overdose of benzocaine (see Institutional Review Board Statement). From each fish, the gills were dissected out and incubated in a PBS-antibiotic mix buffer solution (200 U mL^−1^ penicillin, 200 μg mL^−1^ streptomycin and 400 μg mL^−1^ gentamicin) for 45 min at 17 °C. The samples were enzymatically disrupted with 1 mg mL^−1^ collagenase (type I) in fresh PBS-antibiotic mix buffer. Then, the cell suspension was passed through a 100 μm^2^ cell strainer and centrifuged at 1200× *g* for 5 min at 17 °C. The pellet was resuspended in L-15 media supplemented with 5% FBS, 200 U mL^−1^ penicillin and 200 µg mL^−1^ streptomycin. Thereafter, the cells were maintained at 20 °C in 12-well plates (2.5 × 10^5^ cells per well). The following day, the primary cultures of gill cells were treated with either 10 µg mL^−1^ LPS or 30 µg mL^−1^ poly I:C in FuGENE^®^ using the same induction periods used for RTgill-W1 cells.

### 2.4. In Vivo Fish Experiments

Healthy rainbow trout fingerlings (6 ± 2 g, n = 108) were obtained from the Río Blanco fish farm (see Institutional Review Board Statement). The fish were acclimated for 7 days in 200 L plastic tanks containing aerated freshwater (refreshed every alternate day to eliminate faecal and nitrogenous waste) at 15 ± 1 °C. Throughout the experiments, fish were fed daily *ad libitum* with a commercial feed (Skretting, Puerto Montt, Chile) and exposed to a 12:12 h light–dark photoperiod. After the acclimatization period, and to identify and characterize novel TRIM proteins engaged in the innate immunity of rainbow trout, a bacterial challenge with *F. psychrophilum* CC5 was performed (anticipating a 10% mortality rate in fish) [35]. The bacterium was recovered from rainbow trout in 2014 and authenticated through conventional phenotyping and 16S rDNA-based PCR [36,37]. To produce the bacterial inoculum that was administered to the fish, TYES agar plates (0.4% tryptone, 0.05% yeast extract, 0.02% anhydrous calcium chloride, 0.05% magnesium sulphate heptahydrate and 1% bacteriological agar) was used at pH 7.2. This medium was initially prepared in a solid state for the first bacterial growth, ensuring not more than two sub-cultured growths from stock cultures. However, it was later used in liquid state (without bacteriological agar) at aerobic agitation (15 °C, 100 rpm for 3–5 days) [38].

The challenge was performed considering two groups (control and *F. psychrophilum*) in duplicated 6 L plastic tanks (with 27 fish each one). The tanks had aerated dechlorinated freshwater at 15 ± 1 °C, and similar to what was described above, the water was changed every two days in each tank to remove faecal and nitrogenous waste. Regarding fish, they were anesthetized using a benzocaine solution (30 mg L^−1^) and subsequently inoculated by intramuscular injection with 0.1 mL of TYES broth (control group) or 0.1 mL of 5.6 × 10^5^ CFU fish^−1^ (challenge group). The duration of the experiment was 30 days with daily monitoring for disease symptoms. Deceased fish (which were excluded from analysis samples), were removed daily from each tank and analysed by direct streaking of several samples (i.e., skin lesion, kidney, liver and spleen) onto TYES agar plates that were incubated at 15 °C for 5 days. Biochemical and PCR analyses were conducted on isolates to validate whether the injected bacterium was the mortality cause. Additionally, to preserve tank density, an equivalent number of fish were randomly removed from the non-infected tank whenever a fish inoculated with the *F. psychrophilum* CC5 isolate was removed.

Fish samples were taken from all groups over several days following infection (1, 2, 4, 6, 8, 10, 15 and 30 days). At each sampling, three individuals were sampled per tank. For this, fish were first anesthetized using a benzocaine solution (30 mg L^−1^) and subsequently euthanized with an overdose of the same solution (100 mg L^−1^). Then, a section of the second gill tissue was collected, instantly frozen in liquid nitrogen and stored at −80 °C for later processing.

### 2.5. Molecular Cloning and Sequencing

PCR products were gel purified and sequenced as previously described by Álvarez et al. [33]. Briefly, amplicons were isolated with an E.Z.N.A.^®^ Gel Extraction Kit (Omega Biotek, Norcross, GA, USA) and cloned using the TOPO TA with the pCR 2.1 TOPO^®^ vector (Thermo Fisher Scientific). Plasmids were transformed into DH5α *Escherichia coli* cells and purified using an E.Z.N.A.^®^ Plasmid Mini Kit I (Omega Biotek). Plasmid constructs were verified by PCR using a GOTaq polymerase (Promega) and an ESCO^®^ Aerins™ thermal cycler (Horsham, PA, USA). Thereafter, the sequences were obtained by pyrosequencing under the directions and services provided by Macrogen Inc. (Seoul, Korea).

### 2.6. Identification of TRIM CDS in RTgill-W1 and Bioinformatics

To obtain TRIM coding DNA sequences (CDS) presented in this work, we followed the following three different PCR strategies: (1) Specific PCR primers (Table 1) were designed to amplify the entire CDS region of three virus-induced finTRIMs previously described in the spleen of *O. mykiss* [23]. These sequences were arbitrarily named in this study as finTRIM1 (AF483536), finTRIM2 (AM887838) and finTRIM3 (AM887799). (2) Degenerate PCR primers were produced to non-specifically amplify any TRIM belonging to the C-IV family (currently the group of TRIM proteins with the highest number of members associated with immunological activity in higher vertebrates) [39]. For this, two forward primers were designed in the RING/B-box N-terminal domain and two reverse primers were designed in the PRYSPRY (B30.2) C-terminal domain (Table 2). (3) Additional TRIM CDS were obtained through an EST contig sequence search in *O. mykiss* using the GenBank database.

A bioinformatic approach was performed to determine the identity at protein level of each sequence using the protein database UniProt. To confirm the presence of the main domains of TRIM proteins, the Conserved Domain Database (NCBI) was used along with a prediction of the secondary structure using JPred4 server [40]. Comparison between sequences were performed by multiple sequence alignments with Clustal Omega (Hinxton, UK) and analysed with Jalview [41]. Phylogenetic trees were obtained by the Maximum-Likelihood (ML) method provided by MEGAX (https://www.megasoftware.net, accessed 21 December 2025) with bootstrap test (1000 replicates). Finally, three-dimensional structures templates were obtained from the Protein Data Bank database.

### 2.7. RNAi Gene-Silencing Assay

Based on the gene expression results and to verify the involvement of OmTRIM25 in the immune response of rainbow trout against LPS (at *in vitro* level), an RNA-mediated interference (RNAi) platform was used to silence OmTRIM25 expression (as previously demonstrated) [42,43]. Briefly, small interfering RNA (siRNA) were designed and obtained from Integrated DNA Technologies (Coralville, IA, USA) using the Custom Dicer-Substrate siRNA Tool. Then, RTgill-W1 cells were pre-incubated with either 50 nM of two sets of OmTRIM25-siRNA or GFP-siRNA (technique control) in FuGENE^®^ for 14 h (Table 3). Thereafter, the L15-media was replaced with fresh media containing 10 µg mL^−1^ LPS and incubated for 8 h. The dose and duration of treatment of siRNA were chosen based on previous publications using RNAi platforms in different fish cell lines [44,45].

### 2.8. Total RNA Extraction, cDNA Synthesis and Reverse Transcription Quantitative Real-Time PCR (RT-qPCR)

Total RNA was isolated using the TRIzol^®^ reagent and the RNA E.Z.N.A.^®^ Total RNA Kit I (Omega BioTek). Briefly, samples were analysed using TRIzol^®^ (Thermo Fisher Scientific) and transferred into the kit filter tubes to proceed with the manufacturer instructions. RNA concentration was measured using a ND-1000 spectrophotometer. Then, isolated RNA was reverse transcribed into cDNA using the AffinityScript cDNA Synthesis kit (Agilent Technologies, Santa Clara, CA, USA) and the ESCO^®^ Aerins™ thermal cycler.

For RT-qPCR, the cDNA amplification (10 ng cDNA per reaction) was performed using Brilliant II SYBR^®^ Green QPCR Master Mix (Agilent Technologies) in Mx3000P qPCR System (Agilent Technologies). Each sample was analysed in duplicate for both target gene and reference gene (GAPDH). The expression values were calculated through the 2^−ΔΔCt^ method [46]. Regarding the specific qPCR primers (Table 4), they were designed using AmplifX v1.7.0 (adjusting the parameters for an annealing temperature of 60 °C, as well as an expected amplicon size between 90 and 200 bp). The absence of primer dimers and unwanted secondary structures was verified by OligoAnalyzer™ (Integrated DNA Technologies). Finally, IDT Integrated DNA Technologies synthesized the selected primers.

### 2.9. Statistical Analysis

Statistical analysis (e.g., means, standard deviations, normal distribution analysis by Shapiro–Wilk test) and heatmaps were performed using GraphPad Prism 8.01. Moreover, data from cytokines and TRIM gene expression were analysed by multiple *t*-tests against the experimental control group (within each time point) using the same software. Regarding correlation analysis of gene expression, this was carried out by Corrplot in Rstudio (v.2023.03.0 Build 386). On the other hand, knock-down results were analysed using one-way ANOVA and Tukey’s multiple comparison test (after Shapiro–Wilk test). In all the analyses, differences between data groups were considered statistically significant when *p*-value was <0.05.

## 3. Results

### 3.1. Novel TRIM-like Sequences Identified in RTgill-W1 Cells

Both sequences corresponding to finTRIM1 and finTRIM2 were isolated from RTgill-W1 cells. However, finTRIM3 expression was not detected under the PCR conditions used. In addition, two TRIM-like transcripts corresponding to human orthologues, named OmTRIM25 and OmTRIM16, were isolated and sequenced by the non-specific PCR amplification method. Finally, the EST contig sequence search identified other two TRIM orthologues, named OmTRIM8 and OmTRIM62. All the novel transcripts found (not including the finTRIMs already published) were submitted and registered in the Nucleotide NCBI database as RTgill-W1 novel TRIM-like mRNA sequences (Table 5).

**Figure 1:**
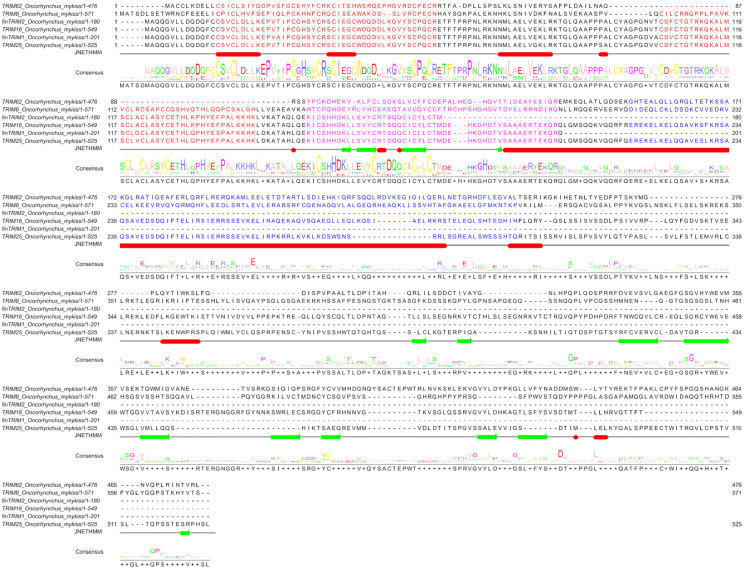
Sequence alignment (by Clustal Omega) between TRIM-like proteins found in RTgill-W1 cells. The main domains of the RBCC motif are highlighted in colours: RING = orange; B-box 1 = red; B-box 2 = purple; coiled-coil = blue.

The multiple alignment of the predicted amino acid sequences showed a high conservation in the N-terminus regions throughout all the OmTRIMs and finTRIMs proteins that were found (Figure 1). Moreover, a search in the Conserved Domains Database (NCBI) revealed that all sequences display the presence of the RBCC motif as a structural signature of the TRIM superfamily protein, including the RING, B-box and coiled-coil.

**Figure 2:**
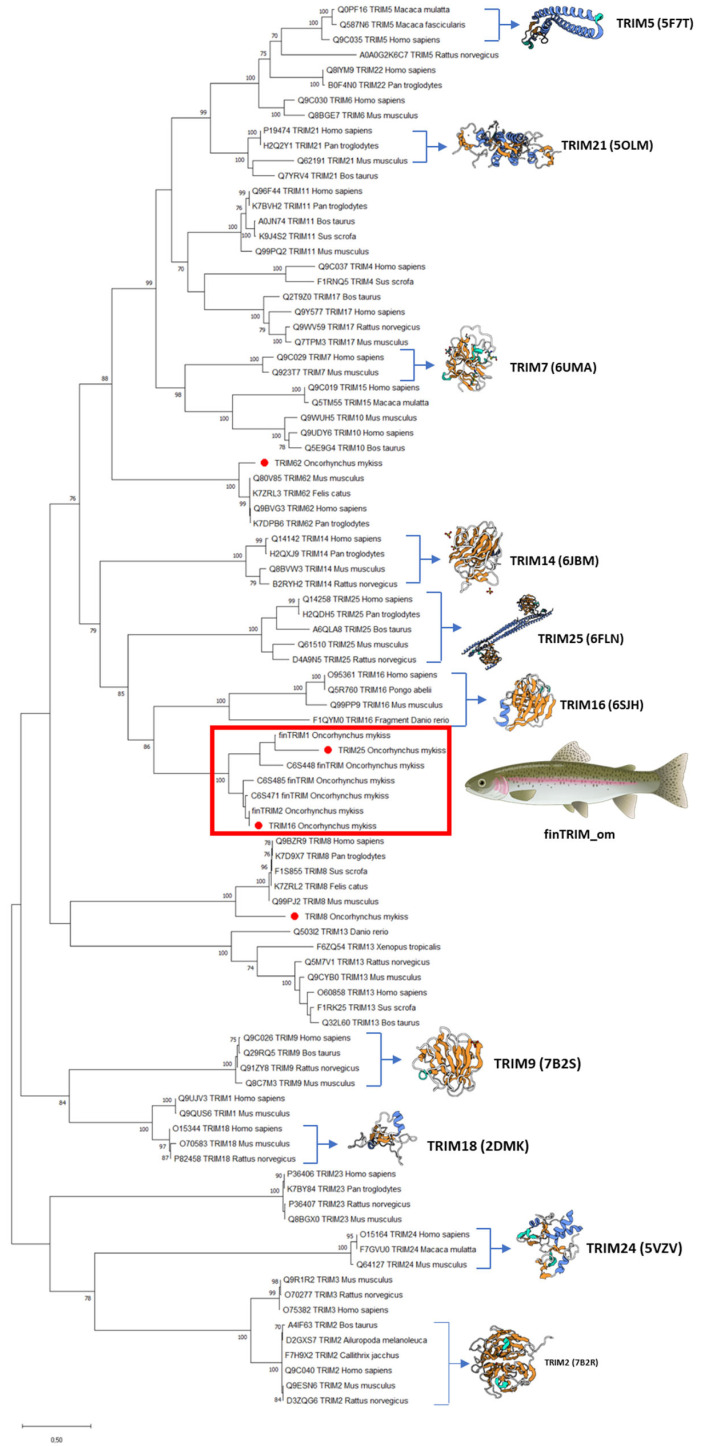
Phylogenetic tree grouping the TRIM-like proteins identified in RTgill-W1 cells. An unrooted phylogenetic tree of TRIM proteins from fish and other species was produced using an amino acid multiple alignment and the ML method within the software MEGAX (https://www.megasoftware.net, accessed 21 December 2025). The percentage of replicate trees (in which the associated taxa clustered together in a bootstrap test with 1000 replicates) is shown next to the branches. Novel TRIMs are highlighted with red dots.

Regarding the phylogenetic analysis, data showed that OmTRIM8 and OmTRIM62 were grouped closely to other homologue members, whereas OmTRIM16 and OmTRIM25 were linked to the finTRIM protein family (Figure 2; see red box).

### 3.2. Differential Expression of Novel TRIM-like Genes upon Stimulation

**Figure 3:**
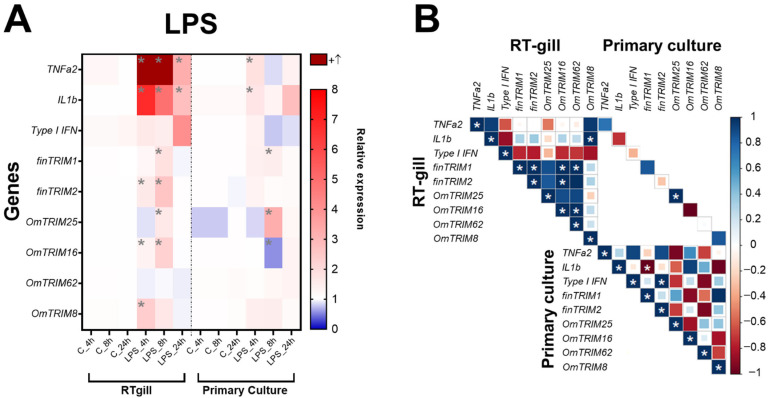
LPS induced the gene expression of cytokines and OmTRIM-like proteins in RTgill-W1 cells and primary gill cultures (in a time dependent manner). RTgill-W1 cells and primary gill cultures were stimulated with 10 µg mL^−1^ LPS for up to 24 h. (**A**) Gene expression of cytokines (TNF-α2, IL-1β and type I IFN) and OmTRIM-like proteins was quantitatively measured using RT-qPCR at 4, 8, and 24 h post-LPS treatment. Results were expressed as the mean ± SEM of six experimental replicates (*: *p* < 0.05 compared to control group). (**B**) Correlation between LPS-induced transcripts in RTgill-W1 cells and primary gill cultures (*: *p* < 0.05).

In RTgill-W1 cells, LPS was able to significantly induce the expression of TNF-α2 and IL-1β at 4, 8 and 24 h post-stimulation (Figure 3A). Furthermore, the same cell model showed that most of TRIM-like genes were up-regulated at 8 h (LPS treatment), except for OmTRIM8 and OmTRIM62. Moreover, OmTRIM8 was specifically up-regulated at 4 h only. Interestingly, the expression of some TRIM transcripts positively correlated between them. For instance, finTRIM1 was correlated with finTRIM2, while OmTRIM16 was correlated with OmTRIM62. In addition, finTRIM 2 was positively correlated with OmTRIM16 and OmTRIM16 with OmTRIM62 (Figure 3B). OmTRIM25 was the only protein whose expression correlated between RTgill-W1 cells and primary gill cultures.

**Figure 4:**
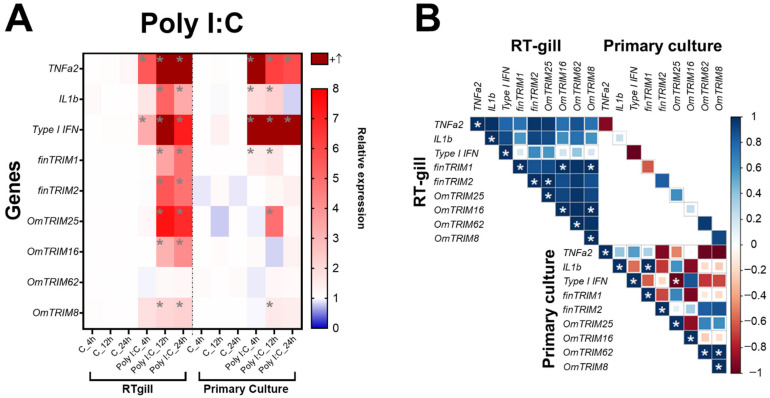
Poly I:C induced the gene expression of cytokines and OmTRIM-like proteins in RTgill-W1 cells, as well as in primary gill cultures (in a time dependent manner). RTgill-W1 cells were stimulated with 30 µg mL^−1^ poly(I:C) for up to 24 h. (**A**) Gene expression of cytokines (TNF-α2, IL-1β and type I IFN) and OmTRIM-like proteins was quantitatively measured using RT-qPCR at 4, 12, and 24 h post-poly(I:C) treatment. Results were expressed as the mean ± SEM of six experimental replicates (*: *p* < 0.05 compared to Control groups). (**B**) Correlation between poly I:C-induced transcripts in RTgill-W1 cells and primary gill cultures (*: *p* < 0.05).

In comparison, poly I:C stimulus caused a significant modulation of pro-inflammatory cytokines such as TNF-α2 and type I IFN (both RTgill-W1 cells and primary gill cultures) at all-time points, while IL-1β was induced at 12 and 24 h only in RTgill-W1 cells (Figure 4A). In comparison, the stimulus with poly I:C caused a higher expression of TRIM-like transcripts than LPS (in RTgill-W1 cells), but in the later time points (12 and 24 h post-stimulation). Notably, OmTRIM16 and OmTRIM62 were not modulated by LPS or poly I:C at any time point of evaluation. Regarding the correlation analysis, the results showed that almost all the molecules induced by poly I:C are positively correlated, but only few comparisons reached significance, including finTRIM1 with OmTRIM16 and OmTRIM8 and OmTRIM16 with OmTRIM8 (Figure 4B).

In primary cultures, poly I:C stimulation was significantly more prone to induce an immune response than LPS. Indeed, poly I:C produced a significant upregulation of type I IFN and TNF-α2 (in every time point examined). On the contrary, LPS only induced IL-1β after 24 h post-stimulation (Figure 3A).

In terms of TRIM expression, both LPS and poly I:C induced the expression of OmTRIM25 only at 8 and 12 h of stimulation, respectively (Figure 3A and Figure 4A). furthermore, only a few correlations were detected in primary cultures. For example, IL-1β negatively correlated with finTRIM1 under LPS stimulation, while poly I:C treatment produced the opposite effect, with a positive correlation between IL-1β and finTRIM1 (Figure 3B and Figure 4B).

### 3.3. Rainbow Trout Infected with a Bacterial Pathogen Showed a Time-Dependent Expression of TRIMs in Gill Tissue

**Figure 5:**
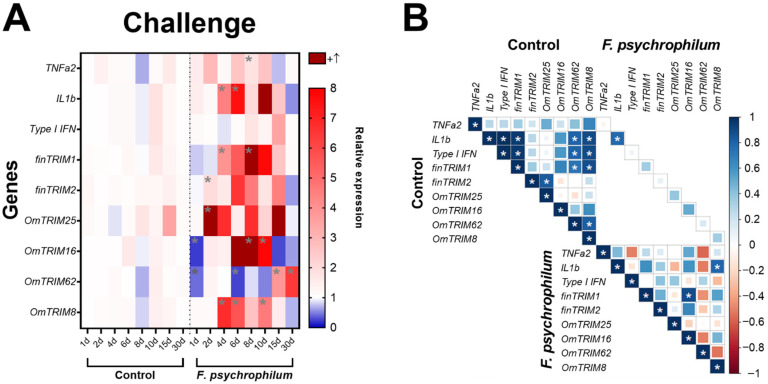
Infection with *F. psychrophilum* CC5 induced the gene expression of OmTRIM-like proteins in rainbow trout. Animals were infected intramuscularly with a LD_50_ of *F. psychrophilum* and gill tissue samples were analysed in a period of 30 days. (**A**) gene expression of the cytokines TNF-α2, IL-1β and type I IFN and OmTRIM-like proteins was quantitatively measured using RT-qPCR at different time points. Results were expressed as the mean ± SEM, n = 3 (*: *p* < 0.05 vs. Control groups). (**B**) Correlation between *F. psychrophilum*-induced transcripts in rainbow trout gills (*: *p* < 0.05).

After the challenge with *F. psychrophilum* CC5, the analysis of cytokine expression (Figure 5A) revealed that the bacteria significantly up-regulated TNF-α2 (at day eight) and IL-1β at days four and six). Interestingly, TRIM transcripts showed different patterns of expression. For instance, OmTRIM25 and finTRIM2 were significantly up-regulated earlier (after two days of infection) and then finTRIM 1 and OmTRIM8 had peaks of expression from the fourth day post-infection. Moreover, OmTRIM16 significantly increased its expression after eight days, and this increase was maintained later as well at day ten. Finally, OmTRIM62 had expression peaks in the last days of analysis (at 15 and 30 days of infection). Although basal levels of gene expression are positively correlated between different molecules, we detected only a significant correlation between IL-1β and OmTRIM8 and between finTRIM 1 and OmTRIM16 (Figure 5B).

### 3.4. OmTRIM25 Is Required to Trigger the Expression of TNF-α2 and IL-1β in RTgill-W1 Cells During LPS Stimulation

**Figure 6:**
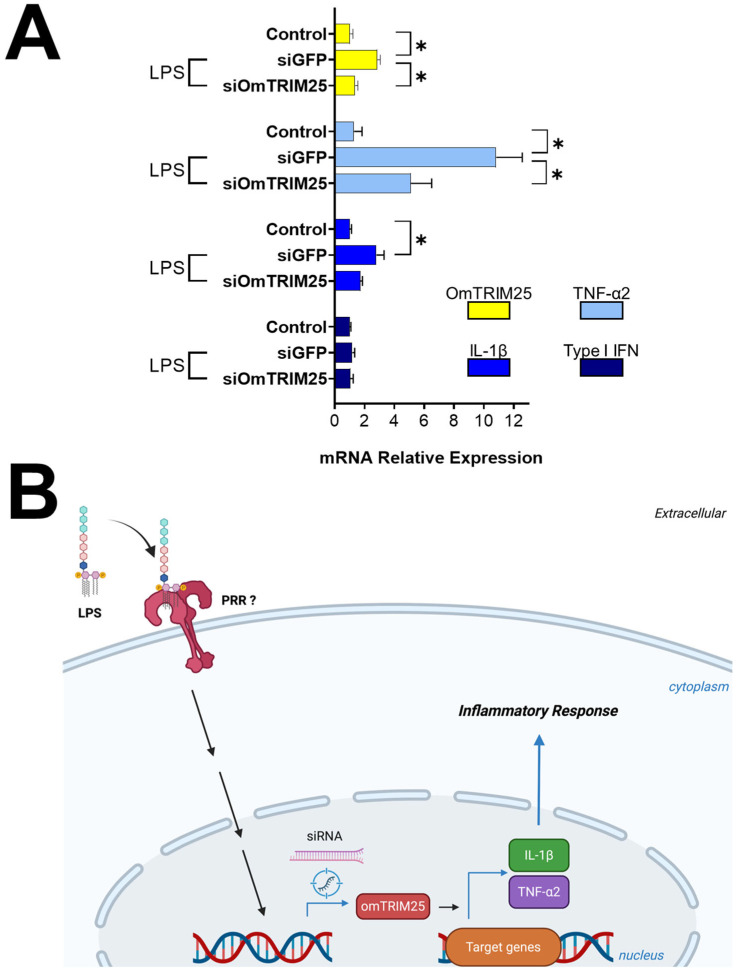
LPS-induced inflammatory response in RTgill-W1 cells mediated by OmTRIM25. (**A**) RTgill-W1 cells were incubated with 50 nM siOmTRIM25 for 14 h and then stimulated with 10 µg mL^−1^ LPS for 8 h. The gene expression of OmTRIM25 and cytokines (TNF-α2, IL-1β and type I IFN was quantitatively measured using RT-qPCR). Results were expressed as the mean ± SEM of six experimental replicates (*: *p* < 0.05). (**B**) Schematic pathway representing the proposed mechanism of action of OmTRIM25.

After 8 h treatment with LPS, cells pre-incubated with siGFP displayed a significant upregulation of OmTRIM25, TNF-α2 and IL-1β, but not for type I IFN (Figure 6) according to our previous results (Figure 3A). On the other hand, cells pre-incubated with siOmTRIM25 showed a downregulation of OmTRIM25, TNF-α2 and IL-1β. Based on these results, Figure 6B shows a schematic representing the proposed mechanism of action of OmTRIM25.

## 4. Discussion

Across vertebrates, increasing attention has been paid to understanding the molecular and cellular evolution of host defence systems [2,4]. To contribute to the knowledge of this topic, the current study focused on the *in vitro*/*in vivo* identification and characterization of novel TRIM proteins involved in the innate immune response from rainbow trout.

In fish, gills are a specialized organ with a respiratory function. However, they also contain mucosa-associated lymphoid tissue (MALT), which is in continuous contact with the external environment and potential pathogens [47]. Thus, gills are a target to characterize immunological mechanisms. In addition, supporting the idea that TRIM proteins are conserved throughout the metazoans [10], our data showed the identification of both human TRIM orthologues and finTRIMs (e.g., OmTRIM8, OmTRIM16, OmTRIM25, OmTRIM62, finTRIM1 and finTRIM2), as well as their expression patterns in RTgill-W1 cell line, primary gill cultures and in gills from infected rainbow trout. Among some of the detected TRIM proteins (e.g., TRIM8, TRIM25, TRIM62), which have previously been linked to the immune system in other organisms [18,19,25,28,29,30,31,48,49,50,51,52], TRIM62 was highly up-regulated after the challenge with LPS or poly I:C (in RTgill-W1 cells and primary gill cultures) along with the expression of pro-inflammatory cytokines such as TNF-α2, IL-1β and type I IFN. This suggests that TRIM members may be involved in the local immune response of the gill. It is also worth mentioning that the expression of TRIM proteins was significantly higher in cells treated with poly I:C than LPS, indicating increased sensitivity to viral infection. Also, these results support the evidence proposing the antiviral role of the TRIM protein family. For instance, TRIM proteins can be implicated during the inhibition of the viral life cycle [13]; the modulation of retinoic acid inducible gene I (RIG-I) [53]; and the regulation of the signalling cascade after TLR3 activation [54]. Additionally, TRIM2b (from zebrafish) has been reported to promote the degradation of viral RNAs from Spring viremia of carp virus (SVCV), a lethal freshwater pathogen of cyprinid fish [55]. Even more, Zhang et al. [56] reported that TRIM23 and TRIM32 from *O. mykiss* decrease the copy numbers of the Infection Haematopoietic Necrosis virus (IHNV). Nevertheless, more research is needed to determine the role of novel TRIM members in the immunological pathways of fish under *in vivo* viral challenges.

Although we found correlated gene expression patterns among TRIM proteins after LPS stimulation, currently there is no evidence supporting the possible involvement of TRIM proteins in antibacterial responses in rainbow trout, it remains unclear whether these relationships are mutually dependent, synergistic or independent in terms of signalling and mechanisms. To improve knowledge about this, and based on our *in vitro* data, despite a higher TRIM expression observed in cells stimulated with poly I:C (compared with those treated with LPS), the subsequent experiments focused on an *in vivo* experiment with fish challenged with *F. psychrophilum* as a pathogenic model. This may contribute to understanding the role of TRIM proteins in bacterial infections, which is relevant due to the high sanitary relevance of bacterial diseases in the Chilean salmonid industry [57]. *F. psychrophilum* is a Gram-negative bacterium responsible for rainbow trout fry syndrome and bacterial cold-water disease [58]. Indeed, we detected the upregulation of TRIM proteins at different time points after the challenge. This could be related with pro-inflammatory and anti-inflammatory molecules that are modulated during an infection (reaching their peak expression at different stages depending on their function) [59,60]. For example, OmTRIM25 and finTRIM2 were significantly up-regulated after two days of infection, while IL-1β and TNF-α2 reached their peaks of expression at four and six days post-infection, respectively. These results shed light on the potential involvement of OmTRIM25 and finTRIM2 in the modulation of bacterial pattern recognition with direct effects linked to the expression of cytokines and even for other TRIM proteins that we found expressed at later time points. Moreover, this hypothesis can be supported by the previous literature pointing out the role of TRIM proteins in modulating immune signalling pathways [61,62]. On the other hand, the expression peak of OmTRIM62 (15 and 30 days post-infection) suggests a possible implication in long-term mechanisms (e.g., adaptive immune response). Accordingly, the role of TRIM proteins in adaptive immunity has been noted to be crucial for T cell differentiation and production of polarizing cytokines [63,64]. However, the ability of TRIM62 to regulate adaptive immunity in fish has not been confirmed yet.

Considering the modulation of OmTRIM25 after the challenge with LPS (found in RTgill-W1 cells and primary gill cultures) and the early expression of this TRIM protein in gills from infected rainbow trout with *F. psychrophilum*, we decided to explore more deeply its possible interactions with immune signalling linked to antibacterial defence. Thus, we silenced the expression of OmTRIM25 at a transcriptional level by using siRNA in RTgill-W1 cells stimulated with LPS. Data showed a significant decrease in the expression of OmTRIM25, confirming the efficacy of the gene knock-down. In addition, blocking OmTRIM25 caused a significant downregulation of TNF-α2 and IL-1β, implying the modulatory function of OmTRIM25 over the expression of these cytokines. Although the precise molecular mechanism of how this could occur was not investigated in this study, and additional experiments are necessary to prove the immunomodulatory role of OmTRIM25, the current data open interesting possibilities for future research. For instance, related to ubiquitination process since TRIM proteins are known to possess E3 ubiquitin ligase activity [65,66,67] and such function has been demonstrated for TRIM25 [53,68]. Moreover, TRIM7 can promote the activation of TLR4 (a specific receptor for LPS recognition in higher vertebrates) through its E3 ubiquitin ligase activity [69], and other proteins can regulate IκB levels and modulate the activation of the nuclear factor NF-κB using the same enzymatic activity [70,71,72].

## 5. Conclusions

This study showed that gill tissue expressed novel TRIM proteins that were not previously described in rainbow trout. The results demonstrated that these TRIM transcripts can be induced by a specific viral or bacterial pathogen-associated molecular pattern (at the *in vitro* level) and that they had specific time-dependent expression peaks in gills from rainbow trout after a bacterial infection with *F. psychrophilum* (over a period of 30 days). Furthermore, the findings revealed that OmTRIM25 is needed to trigger LPS-induced expression of pro-inflammatory cytokines (i.e., TNF-α2 and IL-1β) in RTgill-W1 cells, suggesting a potential immunomodulatory function in the gill during bacterial infection.

Overall, the current data provide novel insights into the role of TRIM proteins in rainbow trout immunology and the possible molecular mechanisms that support their function.

## Figures and Tables

**Table 1:** Specific PCR primers to amplify *O. mykiss* finTRIM CDS. F: forward, R: reverse.

Name	GenBank	Primers	Amplicon Size
finTRIM1	AF483536	F: ATGGCTCAACAGGGAGTTCT R: TCACTGCCTCTGTTTCTCAGTC	606 bp
finTRIM2	AM887838	F: ATGGCTCAACAGGGAGTTCT R: TCAATGACTCTTTCTGTTCCCTT	1227 bp
finTRIM3	AM887799	F: ATGGCTCAGCAGGGAGTTT R: CTACAGTTTAACCAGCTCAGCAGTAC	1656 bp

**Table 2:** Degenerate primers to amplify potential C-IV TRIM members.

Primer	Forward (RING)	Reverse (B30.2)
Set1	TGTGGACACASTTACTGYA	AGTYCAGACCACATTCACTSA
Set2	GGMTGCTGGGAYCAGGA	CAGACCACATTCACT

**Table 3:** Designed siRNAs to silence OmTRIM25 gene expression.

siRNA	Sense Sequence	Antisense Sequence
siGFP	rArCrArArCrArGrCrCrArCrArArCrGrUrGrUrArCrArUrCAT	rArUrGrArUrGrUrArCrArCrGrUrUrGrUrGrGrCrUrGrUrUrGrUrArG
siTRIM25-1	rGrCrArGrCrArGrArGrArGrGrArCrUrGrArGrArArArCrAGA	rUrCrUrGrUrUrUrCrUrCrArGrUrCrCrUrCrUrCrUrGrCrUrGrCrArG
siTRIM25-2	rGrGrArGrGrArCrArGrUrGrArUrCrArGrArUrCrUrUrUrACT	rArGrUrArArArGrArUrCrUrGrArUrCrArCrUrGrUrCrCrUrCrCrArC

**Table 4:** Specific primers designed for this study for quantitative RT-qPCR. Melting temperatures (Tm): 60 °C.

Gene	Forward	Reverse	Expected Amplicon Size
finTRIM 1	CTACTGAAGGAGCCGGTGG	CAGTGCAGACATCACACGC	262 bp
finTRIM 2	CTGGACCTGGAGATGTGGCG	TGCAGGAAATTCATAGTGAGGTTT	128 pb
OmTRIM25	TCACCAACTGGTACCAGTTACA	AGAGCACTGGAAACTCCAGGACTT	182 pb
OmTRIM16	AAAGGTGACCTGTACACACCTCT	TCTCTGTTCTGCTGATGTCTTTA	215 pb
OmTRIM62	GATTTCCCGACCTCCAAGTACA	GCAGGTTACCATAGGCTACGAT	166 pb
OmTRIM8	GGAAGTGGAAGTGGCTCTCTAA	TCCATGGTACACACCAGGATCT	116 pb
EF1α	TGGAGACTGGCACCCTGAAG	CCAACATTGTCACCAGGCATGG	127 pb
GAPDH	CCTGCAGAAGGGAATCAAAGTCGT	TCTCATGGGGCTTCATACACTGGA	185 pb
TNF-α2	GTGTGGCGTTCTCTTAATAGCAGC	ATTCCGTCCTGCATCGTTGC	96 pb
IL-1β	GTCACATTGCCAACCTCATCATCG	GTTGAGCAGGTCCTTGTCCTTGA	95 pb
Type I IFN	GATGCTGAGTTTGAGGACAAAGTC	GTTTCATGGCAGGTGATACACAGGA	178 pb

**Table 5:** Novel TRIM-like sequences identified in the RTgill-W1 cell line.

Identified TRIMs in RTgill-W1	GenBank Accession
OmTRIM25	KY073243
OmTRIM16	KY073245
OmTRIM62	KY073247
OmTRIM8	KY073248

## Data Availability

The data presented in this study is available on request from the corresponding author.

## Abbreviations

The following abbreviations are used in this manuscript:

TRIM	Tripartite Motif (TRIM)
LPS	Lipopolysaccharide
Poly I:C	Polyinosinic–polycytidylic acid
dpi	Post-infection
RNAi	RNA interference
RBCC motif	B-box zinc finger domain and a coiled-coil region
finTRIMs	Novel fish TRIMs
RTgill-W1 cells	Cell line from rainbow trout gills
PCR	Polymerase chain reaction
CDS	Coding DNA sequences
ML	Maximum-Likelihood
siRNA	Small interfering RN
RT-qPCR	Reverse transcription quantitative real-time PCR
